# Effects of Prenatal Methcathinone Exposure on the Neurological Behavior of Adult Offspring

**DOI:** 10.2174/1570159X22666240128004722

**Published:** 2024-02-07

**Authors:** Zhang Youyou, Li Zhaoyang, Li Chen, Zhao Shuquan, Wang Hui

**Affiliations:** 1 Department of Geriatric Neurology, The Second Affiliated Hospital of Xi’an Jiaotong University, Xi'an Jiaotong University, No. 157, Xiwu Road, Xi'an, 710004, Shaanxi, P.R. China;; 2 NHC Key Laboratory of Forensic Science, Xi'an Jiaotong University, No. 76, West Yanta Road, Xi'an, 710061, Shaanxi, P.R. China;; 3 Department of Occupational and Environmental Health, School of Public Health, Xi’an Jiaotong University, No. 76, West Yanta Road, Xi'an, 710061, Shaanxi, P.R. China;; 4 Department of Geriatric Neurology, The Second Affiliated Hospital of Xi’an Jiaotong University, Xi'an Jiaotong University, No. 157, Xiwu Road, Xi'an, 710004, Shaanxi, P.R. China;; 5 Department of Forensic Pathology, Zhongshan School of Medicine, Sun Yat-sen University, No. 74, Zhongshan 2^nd^ Road, Guangzhou, 510080, Guangdong, P.R. China

**Keywords:** Methcathinone, prenatal exposure, adult offspring, neurological behavior, anxiety-like behavior, learning and memory ability, 5-hydroxytryptamine, dopamine

## Abstract

**Background:**

Our previous research has shown that prenatal methcathinone exposure affects the neurodevelopment and neurobehavior of adolescent offspring, but the study on whether these findings continue into adulthood is limited.

**Objective:**

This study aims to explore the effects of prenatal methcathinone exposure on anxiety-like behavior, learning and memory abilities, as well as serum 5-hydroxytryptamine and dopamine concentrations in adult offspring.

**Methods:**

Pregnant rats were injected daily with methcathinone between the 7^th^ and 20^th^ days of gestation. The neurobehavioral performance of both male and female adult offspring rats was evaluated by neurobehavioral tests, including open-field tests, Morris water maze (MWM) tests, and novel object recognition (NOR) tests. The levels of 5-hydroxytryptamine and dopamine concentration in rat serum were detected by ELISA.

**Results:**

Significant differences were found in the length of center distance and time of center duration in the open-field test, as well as the times of crossing the platform in the MWM test, between the prenatal methcathinone exposure group and the control group. Results of the NOR test showed that adult offspring rats exposed to methcathinone need more time to discriminate the novel object. No gender differences were detected in the neurobehavioral tests. The serum concentrations of 5-hydroxytryptamine and dopamine in rats exposed to methcathinone prenatally were lower than that in the control group, and the serum dopamine concentration was independent of gender in each group.

**Conclusion:**

Prenatal methcathinone exposure affects the neurological behavior in adult offspring, and 5-hydroxytryptamine and dopamine might be involved in the process.

## 1 INTRODUCTION

Methcathinone, a synthetic cathinone, is among the most widely consumed new psychoactive substances, experiencing a global increase in both usage and popularity [1-3]. Methcathinone was discovered in the late 1920s and was used in the clinical treatment of depression during the 1930s and 1940s [4]. Due to the adverse reactions and addiction to methcathinone, its clinical application is limited. However, it has been widely used as an entertainment drug in many countries and has caused a series of public health problems [5, 6]. Recent data indicates that synthetic cathinones constituted half of the total amount of new psychoactive substances seized in EU Member States in 2021 [7]. Due to its relatively low price and ease of preparation, methcathinone has always been a widely popular drug among young people despite the implementation of a comprehensive and balanced international drug management strategy since the 2000s [8, 9].

Methcathinone exerts potent stimulant and euphoric effects, which may be attributed to the release of dopamine from dopaminergic nerve terminals in the brain. Both *in vivo* and *in vitro* studies have confirmed its neurotoxic effects. Previous population-based studies suggested that methcathinone abuse was associated with various deficits, including impaired problem-solving performance [10], impaired decision-making ability [11], parkinsonian syndrome [12], disrupted reward-effort balance [13], impaired verbal learning and visual memory [14] and other neurofunctional disorders. Methcathinone has effects on body temperature [15], monoamine transporters [16], and motor activity [17, 18] in rats. It could trigger autophagy activation, produce reactive oxygen species production [19] and impair the function of mitochondria's electron transport chain in SH-SY5Y cells [20]. In addition, methcathinone abuse could increase sexual desire and then lead to unwanted pregnancies among young people.

Epidemic data shows a significant increase in drug abuse among women of childbearing age in recent years [21]. Most illegal drugs can cross the placental barrier, and mounting evidence has confirmed that exposure to drugs during pregnancy has a significant impact on the growth and development of both embryos and infants [22, 23]. In our previous study, we conducted a review on the adverse health outcomes in offspring resulting from prenatal methamphetamine exposure. Our findings encompass shorter gestational age, lower birth weight, diminished head circumference, reduced body length in neonates, structural alterations in the brain, impaired cognitive development, motor skills, inhibitory control, and attention in children aged 1 to 15 years [24].

We previously reported that prenatal methcathinone exposure can lead to delayed physical and neural reflex development, as well as impaired learning and memory in offspring of rats from birth to day 42 postpartum [25]. Considering the extensive similarities in chemical structure and neuropharmacological effects between methcathinone and methamphetamine (a well-known neurotoxic drug), as well as the “fetal origin hypothesis of adult diseases” concept postulates since 1990 [26, 27], we speculate that prenatal methcathinone exposure may cause damage to the neurobehavior of adult offspring.

In this study, we aim to explore the effects of prenatal methcathinone exposure on anxiety-like behavior, learning and memory abilities, as well as serum 5-hydroxytryptamine (5-HT) and dopamine (DA) concentrations in adult offspring. This was achieved by assessing neurobehavioral performance through the open-field test, Morris water maze (MWM) test, and novel object recognition (NOR) test in adult offspring rats, followed by detecting the concentrations of 5-HT and DA in rat serum by ELISA.

## 2 METHODS

### 2.1 Animals

Eighteen specific pathogen-free Sprague-Dawley rats, weighing between 200-220 grams, were purchased from Vitalriver Experimental Animal Technology Co. Ltd. (Beijing, China). They were housed in a controlled environment with a 12-hour light/dark cycle, maintaining a temperature of 20-22^o^C and humidity between 40%-50%. The rats had unrestricted access to food and water. Mating of female and male rats occurred at a ratio of 2:1 to produce postnatal rats, with the first day of pregnancy marked as the day the pessary was observed in the ostium vaginae of the female rats. Pregnant SD rats were randomly divided into two groups: the control group (n = 6) and the methcathinone exposure group (n = 6). Methcathinone (CAS: 152610-69-0) was obtained from Wuhan Zhongchang National Research Standard Technology Co.Ltd. (Hubei, China). The administration method and administration time are based on our previous experimental methods [25]. After natural parturition, eight rat pups (gender balanced) from each litter were selected at P0 and then categorized by gender at P21. All animal protocols were conducted in compliance with the Guide for the Care and Use of Laboratory Animals published by the US National Institutes of Health (NIH Publication No. 85-23, revised 1996) and approved by the Xi’an Jiaotong University Animal Welfare Committee.

### 2.2 Neurobehavioral Tests

Four offspring rats (totally gender balanced) from each litter were selected to perform neurobehavioral tests. Then, the 24 rats were randomly divided into 3 groups, ensuring that at least one offspring rat in each litter was assigned to each test. Each offspring rat was only used for one of the 3 neurobehavioral tests.

#### 2.2.1 Open-field Test

The open field test was employed to assess exercise ability and anxiety-like/depression-like behavior during the 12th week. In this test, the offspring rats both in the prenatal methcathinone exposure group and control group were randomly selected, comprising 8 rats in each group and ensuring a gender-balanced distribution. The test room, dark and sound-insulated, underwent a 1-hour acclimatization period for rats, and the equipment was sanitized with 75% ethanol after each trial. Rats were introduced to the center of an open-field arena (100 cm in length, 100 cm in width, and 40 cm in depth) and permitted to explore freely for 5 minutes. A video camera positioned directly above the arena tracked the movement of each rat, and the data were recorded on a computer using ANY-maze software to measure parameters such as distance traveled, speed, moving duration, center distance, center duration, and zone crossings [28]. Spending less time in the center of the arena is indicative of anxiety-like behavior.

#### 2.2.2 Morris Water Maze (MWM) Test

The MWM test was utilized to assess the spatial memory and learning abilities of rats during the 12th week; it consists of acquisition trials on days 1-5 and a spatial probe trial on day 6 [29]. Eight offspring rats were randomly selected from both the prenatal methcathinone exposure group and the control group, ensuring a gender-balanced representation for this test. The specific environmental requirements and experimental methods for the test refer to our previous study [25].

#### 2.2.3 Novel Object Recognition (NOR) Test

NOR test was employed to assess episodic recognition memory in rats during the 12^th^ week. Eight offspring rats were randomly selected from both the prenatal methcathinone exposure group and the control group, ensuring a gender-balanced representation for this test. The experimental apparatus consisted of an open-field box measuring 80 cm in length, 80 cm in width, and 50 cm in depth. The NOR test was performed based on previously described protocols [25, 30], with a two-hour interval between training and testing.

### 2.3 Enzyme-linked Immunosorbent Assay (ELISA)

The rat 5-HT ELISA kit (JYM0326Ra) and rat DA ELISA Kit (JYM0327Ra) were purchased from Wuhan Colorful Gene Biological Technology Co. Ltd. (Hubei, China). Following the neurobehavioral tests conducted on the offspring rats, whole blood was collected, and the samples were left undisturbed at room temperature to allow clotting. Then, centrifuge at 3000 rpm for 20 minutes to remove the clots. The concentration of 5-HT and DA in the serum of 8 rats per group (ensuring a gender-balanced representation) was determined using the sandwich ELISA method.

### 2.4 Statistical Analysis

Results are presented as either mean ± SD (for continuous variable) or median and range (for discontinuous variable). Statistical significance between the two groups was analyzed by two-way analysis of variance (for continuous variable) or the Scheirer-Ray-Hare test (for discontinuous variable) using SPSS 19.0. And *P* < 0.05 is considered to have statistical significance.

## 3 RESULTS

### 3.1 Open-field Test of Adult Offspring

**Fig. (1):**
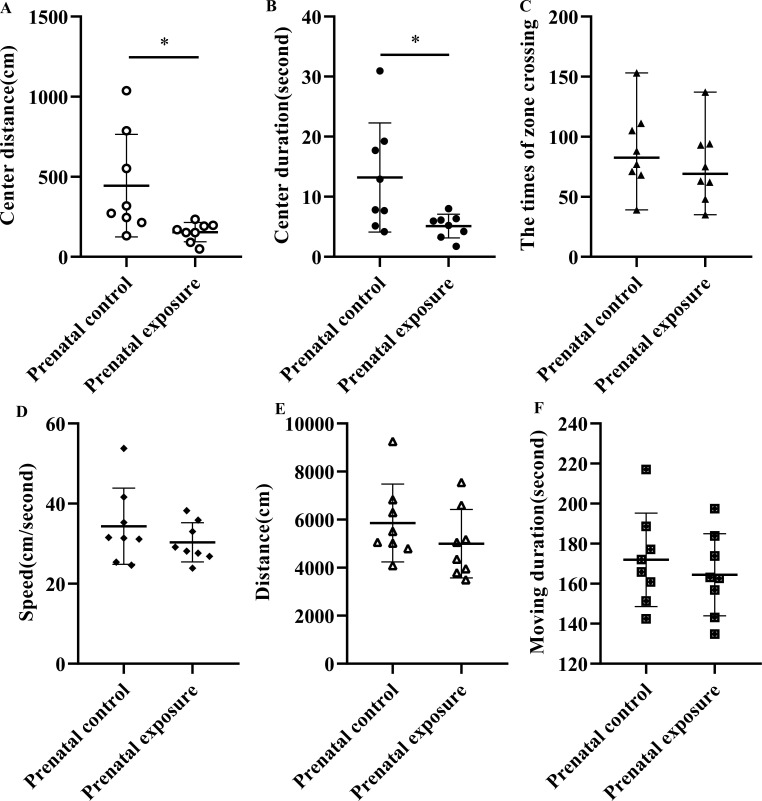
Open-field test in adult offspring rats. The difference in center distance (**A**), center duration (**B**), the times of zone crossing time distance (**C**), speed (**D**), total distance (**E**), and moving duration (**F**) between prenatal methcathinone exposure group and prenatal control group in the open-field test, *P < 0.05.

The results of the open-field test are presented in Fig. (**1**). As depicted in Fig. (**1A**), the length of the center distance of rats in the prenatal methcathinone exposure group (154.5 ± 59.6 cm) was decreased compared with it (444.5 ± 319.7 cm) in the control group (*P* = 0.032) even though the total distance (Fig. **1E**) is unchanged between the two groups (*P* = 0.306). Fig. (**1B**) illustrates that the time of center duration in the prenatal methcathinone exposure group (5.1 ± 2.0 seconds) was significantly shortened compared to the control group (13.2 ± 9.1 seconds) (*P* = 0.041). Other indices from the open field test, including the times of zone crossing (Fig. **1C**), speed (Fig. **1D**), and moving duration (Fig. **1E**), showed a tendency to decrease in the prenatal methcathinone exposure group compared to the control group with no statistical differences. There was no significant main effect of gender observed in either the prenatal methcathinone exposure group or the control group.

### 3.2 MWM Test of Adult Offspring

The results of the MWM test are presented in Fig. (**2**). Fig. (**2A**) indicates that the escape latency in the prenatal methcathinone exposure group was prolonged compared to the control group, with a significant difference observed on day 3 (*P* = 0.004). In Fig. (**2B**), the number of times crossing the platform on the final day was significantly reduced in the prenatal methcathinone exposure group compared to the control group (*P* = 0.018). The results further revealed no gender differences among these indicators.

### 3.3 NOR Test of Adult Offspring

The discrimination index (DI) of the NOR test, representing the ability to discriminate novel objects, was analyzed for each group. A lower DI indicates that rats require more time to distinguish the novel object. As illustrated in Fig. (**3**), the ability of offspring rats in the control group to distinguish new objects is higher than that of rats exposed to methcathinone (*P* = 0 .026). No gender differences were observed in DI.

### 3.4 Serum DA and 5-HT Concentration in Adult Offspring

The concentrations of DA and 5-HT in rat serum were measured by ELISA, as shown in Fig. (**4**). Significant differences were found in the rat serum concentrations of DA (107.3 ± 24.6 pg/ml *vs.* 169.5 ± 14.7 pg/ml) and 5-HT (88.1 ± 11.3 ng/ml *vs.* 139.0 ± 19.1 ng/ml) between the prenatal methcathinone exposure group and the control group (*P <* 0.001). The serum DA concentration was independent of gender in each group (*P* = 0.021).

## 4 DISCUSSION

In our current study, we observed that prenatal exposure to methcathinone led to anxiety-like behavior and impaired learning and memory abilities in adult offspring, as evidenced by the outcomes of the open-field test, MWM test, and NOR test. Additionally, this exposure affected the serum concentrations of DA and 5-HT.

Based on our prior study, we identified impairments in neurodevelopment and learning and memory abilities in adolescent offspring due to prenatal methcathinone exposure [25]. Despite numerous studies highlighting the adverse effects of prenatal drug exposure on adolescents [24, 25], the study on whether these findings continue into adulthood is limited. Therefore, it is crucial to investigate and comprehend the potential long-term consequences of prenatal drug exposure. Notably, prenatal exposure to marijuana has been linked to compromised executive functioning [31] and the development of psychotic symptoms in young adulthood [32], with indirect effects on adult functioning through early initiation of cannabis use [33]. In this study, lower center distance and center duration, prolonged escape latency, decreased platform crossing times and discrimination index were found in adult offspring exposed to methcathinone prenatally. These findings underscore the importance of further research on the enduring impact of prenatal methcathinone exposure on the neurological behavior of offspring.

Cathinones, as β-keto analogs of amphetamines, share pharmacological effects resembling cocaine and amphetamines. However, the neurotoxicity induced by β-keto amphetamines is distinctive despite their chemical structural similarities [34]. Dysregulation of neurotransmitter systems was associated with cathinone-induced neurotoxicity, and previous studies on the effects of cathinones on neurotransmitter systems were mainly focused on the depletion of DA and 5-HT. Methcathinone, as one kind of cathinones, possesses the potential to damage DA and 5-HT neurons [35]. Gygi *et al.* [36] demonstrated decreased DA and 5-HT content after repeated administration of methcathinone in rats. Consistently, our study revealed decreased concentrations of DA and 5-HT in the serum of adult offspring rats.

Dysfunction of neurotransmitters can lead to neurological behavior disorders, including anxiety and impairment of learning and memory ability [37-41]. Jones *et al.* [37] reviewed the classically defined functions of 5-HT that are involved centrally in the control of mood, sleep and anxiety and peripherally in the modulation of gastrointestinal motility. Zarrindast and Khakpai [38] presented evidence highlighting the significant role of dopamine in anxiety modulation across various brain regions. Myhrer [41] conducted a review of neurotransmitter systems implicated in learning and memory, calculating the indirect influential factor of DA and 5-HT on learning and memory in specific behavioral tests, including the water maze, radial maze, passive avoidance, and spontaneous alternation. Importantly, the regulatory role of 5-HT/DA interactions in learning and memory functions has been acknowledged in recent years [39], and it could be further studied by future experimental and clinical studies. Combined with the above finding in this study, we hypothesize that prenatal methcathinone exposure could lead to persistent depletions of DA and 5-HT, subsequently impacting the neurological behavior of adult offspring.

The concept that exposure to abused drugs during pregnancy can induce long-term changes in gene expression and heighten susceptibility to disease in adulthood has been firmly established [42-45]. Crossing the placenta is a necessary condition for an abuse drug to directly affect the developing fetus. The placenta assumes a vital role in fetal programming, acting as a functional interface connecting the mother to the developing fetus throughout pregnancy [46]. The placenta also produces neurotransmitters, including DA and 5-HT, which can circulate and influence brain development [47]. Previous studies [48-50] indicated that 5-HT signaling is associated with the effect of maternal adverse exposure on fetal neurodevelopment. In summary, combined with the findings of our present study, whether prenatal methcathinone exposure affects fetal neurodevelopment *via* disrupts 5-HT/DA synthesized from the placental to the fetal brain needs to be further studied in future research.

## CONCLUSION

In conclusion, prenatal methcathinone exposure is associated with changes in neurological behavior in adult offspring, with the involvement of DA and 5-HT. Further studies should demonstrate the pathways leading to neurological outcomes in adolescent and adult offspring, including memory deficits and psychotic symptoms. Meanwhile, more mechanistic studies are needed to further illustrate potential mechanisms that underlie the link between offspring rats prenatally exposed to methcathinone and the neurological development /behavior from fetal life to adulthood.

## Figures and Tables

**Fig. (2):**
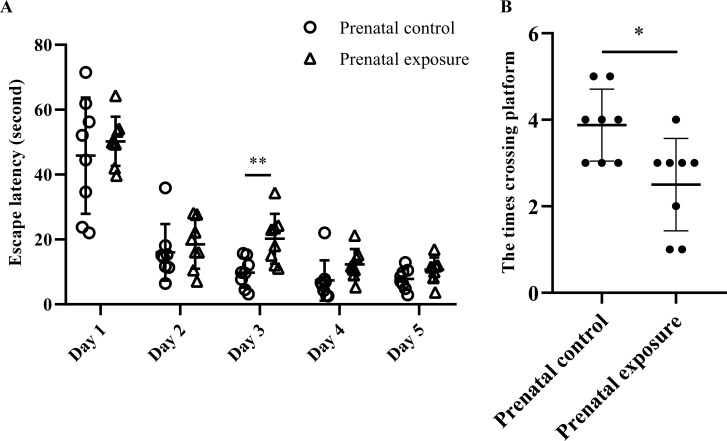
Morris water maze test in adult offspring rats. A, The difference in escape latency between the prenatal methcathinone exposure group and prenatal control group on the training days (**P < 0.01); B, The difference of the times crossing the platform on the last day between prenatal methcathinone exposure and control (*P < 0.05).

**Fig. (3):**
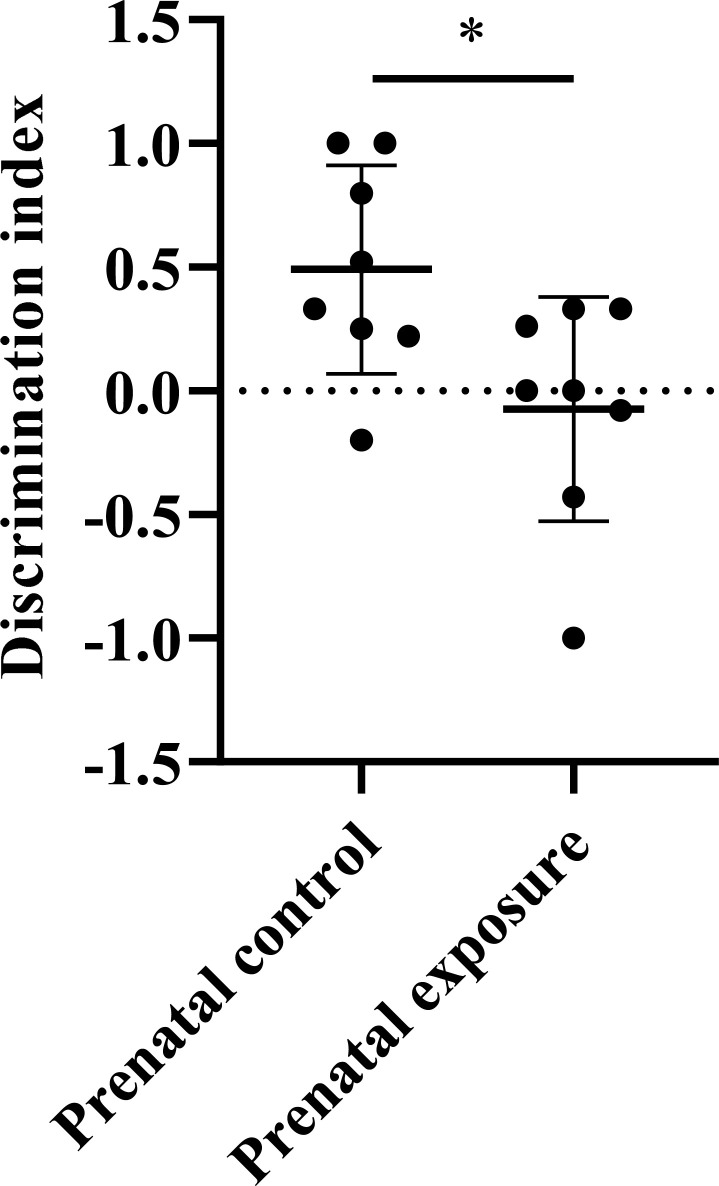
Novel object recognition test in adult offspring rats. The difference in the discrimination index in the NOR test between the prenatal methcathinone exposure group and prenatal control group (*P < 0.05).

**Fig. (4):**
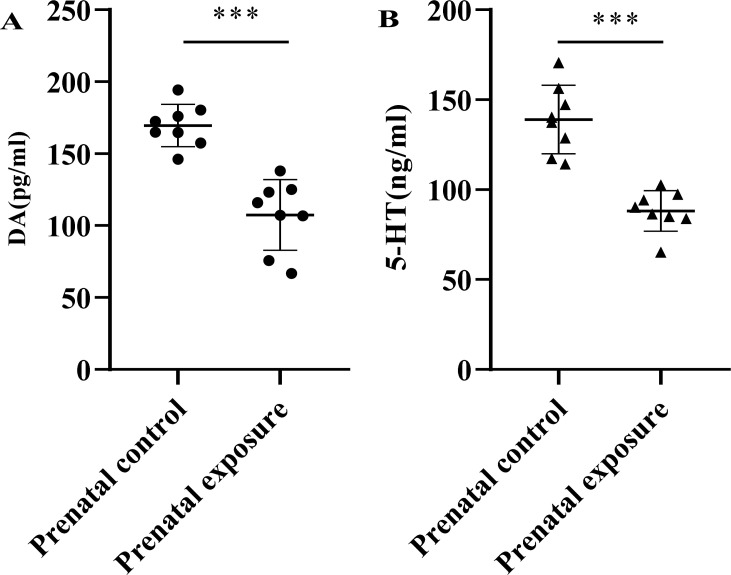
Effects of prenatal methcathinone exposure on the serum concentration of dopamine and 5-Hydroxytryptamine in adult offspring rats. The difference in the serum concentration of DA and 5-HT dopamine between the prenatal methcathinone exposure group and prenatal control group (***P < 0.001).

## Data Availability

The data that support the findings of this study are available from the corresponding author (ZY), upon reasonable request.

## LIST OF ABBREVIATIONS

DA: Dopamine
DI: Discrimination Index
ELISA: Enzyme-linked Immunosorbent Assay
5-HT: 5-hydroxytryptamine
MWM: Morris Water Maze
NOR: Novel Object Recognition
